# Artificial Intelligence Empowers Solid-State Batteries for Material Screening and Performance Evaluation

**DOI:** 10.1007/s40820-025-01797-y

**Published:** 2025-06-06

**Authors:** Sheng Wang, Jincheng Liu, Xiaopan Song, Huajian Xu, Yang Gu, Junyu Fan, Bin Sun, Linwei Yu

**Affiliations:** 1 School of Future Science and Engineering, Soochow University, Suzhou, 215222 People’s Republic of China; 2 School of Electronics Science and Engineering, Nanjing University, Nanjing, 210023 People’s Republic of China; 3 School of Electronics Science and Engineering, Soochow University, Suzhou, 215006 People’s Republic of China

**Keywords:** Solid-state batteries, Artificial intelligence, Deep learning, Material screening, Performance evaluation

## Abstract

Solid-state batteries are widely recognized as the next-generation energy storage devices with high specific energy, high safety, and high environmental adaptability. However, the research and development of solid-state batteries are resource-intensive and time-consuming due to their complex chemical environment, rendering performance prediction arduous and delaying large-scale industrialization. Artificial intelligence serves as an accelerator for solid-state battery development by enabling efficient material screening and performance prediction. This review will systematically examine how the latest progress in using machine learning (ML) algorithms can be used to mine extensive material databases and accelerate the discovery of high-performance cathode, anode, and electrolyte materials suitable for solid-state batteries. Furthermore, the use of ML technology to accurately estimate and predict key performance indicators in the solid-state battery management system will be discussed, among which are state of charge, state of health, remaining useful life, and battery capacity. Finally, we will summarize the main challenges encountered in the current research, such as data quality issues and poor code portability, and propose possible solutions and development paths. These will provide clear guidance for future research and technological reiteration.

## Highlights

- The latest advancements in the application of machine learning (ML) for the screening of solid-state battery materials are reviewed.
- The achievements of various ML algorithms in predicting different performances of the battery management system are discussed.
- Future challenges and perspectives of artificial intelligence in solid-state battery are discussed.

## Graphical abstract

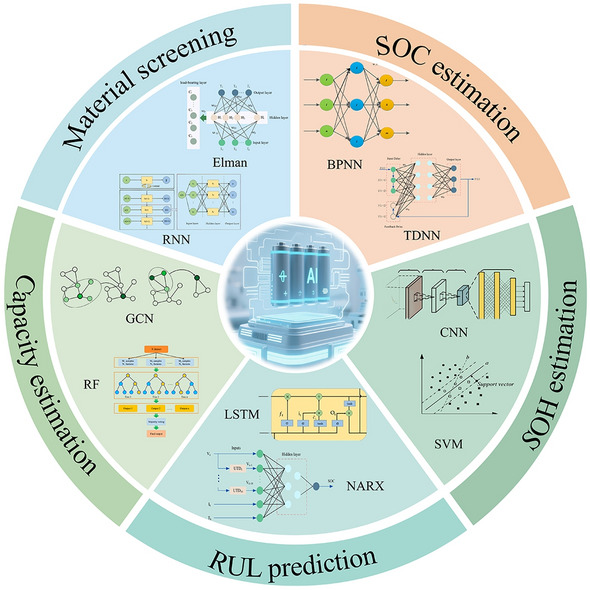

## Introduction

In the context of the global community actively seeking sustainable energy solutions [1–3], transformation of the energy structure is of decisive significance for alleviating the energy crisis, reducing environmental pollution, and promoting sustainable economic development. As a crucial force in achieving low-carbon emissions reduction and addressing energy challenges, electric vehicles are experiencing rapid development at an unprecedented pace [4–6]. Solid-state batteries, with advantages in energy density, safety, and cycle life over traditional lithium-ion batteries, have become a focus of next-generation energy storage devices [7–12]. Although solid-state batteries hold immense potential, their complex chemical environments necessitate novel approaches to overcome material and performance challenges. Meanwhile, the development momentum of artificial intelligence (AI) technology has shown an explosive growth trend and has already played a powerful driving role in various different fields [13–20]. Machine learning (ML), deep learning (DL), etc., as the core branches of AI, possess the astonishing ability to process massive amounts of data [21]. Through complex algorithm models, they can excavate the hidden complex patterns, laws, and trends behind the data and, based on these findings, make highly accurate predictions and intelligent decisions, thus enabling the screening of solid-state battery materials and the prediction of their performance.

Although the future of solid-state batteries is promising, there are still many challenges in their journey toward practical applications. In the aspect of material screening, identifying an ideal combination of solid-state electrolytes (SSEs) and electrode materials demands a comprehensive and profound exploration and screening of a voluminous and diverse material system [22]. Each step in experimental design, sample preparation, and performance characterization requires significant time and financial resources. Traditional trial-and-error methods are often slow and inefficient, hindering their ability to keep pace with rapidly evolving technologies [23]. However, the application of AI and ML models has constructed an efficient and precise strategic system for screening SSEs and electrode materials, which significantly enhances the efficiency and success rate of material screening and substantially shortens the research and development cycle [24–26]. For example, Ahmad et al. [27] computationally screened over 12,000 inorganic solids for next-generation lithium-metal anode batteries. Using a ML model, they predicted new SSEs’ mechanical properties, and cross-validation verified the model’s robustness. In another study, Hajibabaei et al. [28] employed an extensible sparse Gaussian process regression form and replicated the experimental melting temperature and glass-crystallization temperature of Li_7_P_3_S₁₁ and conducted a simulation analysis of the lithium diffusion rate. As a result, an unknown phase with a low lithium diffusion rate, which should be avoided, was discovered. AI algorithms not only speed up material development but also predict solid-state battery key performance indicators. For example, Zahid et al. [29] proposed a state-of-charge (SOC) estimation method based on a neuro-fuzzy system with subtractive clustering. The results indicate that the proposed model exhibits high accuracy, with a maximum estimation error of less than 0.1%. This establishes the foundation for real-time adaptive battery management systems (BMS) with dynamic performance optimization capabilities.

The BMS is a system that conducts intelligent management and maintenance of batteries. However, the accurate determination and prediction of core indicators such as the SOC [30], state of health (SOH) [31], remaining useful life (RUL) [32], and battery capacity have always been formidable challenges that traditional research methods struggle to overcome. Traditional methods, such as the equivalent circuit model (ECM) and physical model (PBM), are constrained by the complex electrochemical processes, the variable operating conditions (such as temperature variations, fluctuations in charge-discharge rates, and mechanical stresses), and the dynamic changes in the internal physical and chemical properties of the battery during long-term use [33, 34]. These methods exhibit limitations when dealing with these issues. However, AI is able to undertake systematic learning and in-depth analysis of a substantial volume of battery operation data by virtue of its formidable data processing capabilities and intelligent analytical algorithms. It can thereby establish highly accurate evaluation models, offering a reliable basis for the BMS. This effectively guarantees the safe and efficient operation of the battery and significantly expedites the progression of solid-state batteries from theoretical concepts to practical applications. For example, Sahinoglu et al. [35] proposed a novel method for estimating the SOC of lithium-ion batteries based on ML. The results demonstrated that this method has more advantages compared to advanced techniques such as support vector machine (SVM), relevance vector machine (RVM), and neural network (NN), with the root-mean-square error (RMSE) and mean absolute error (MAE) being less than 0.14% and 0.36%, respectively.

**Fig. 1:**
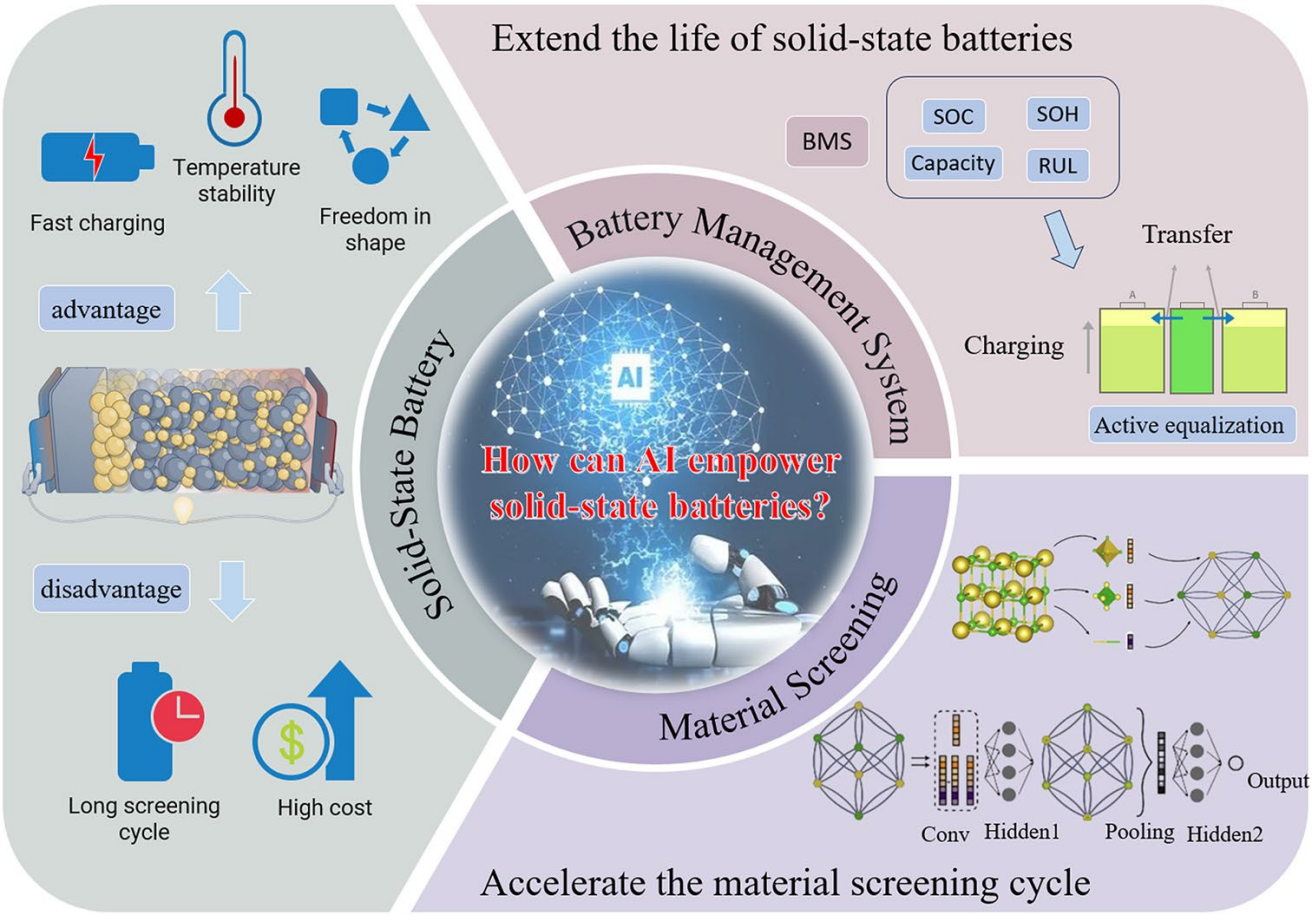
AI is applied in two aspects of solid-state batteries, material screening and performance evaluation. Solid-State Battery [41]. Copyright (2023) Spring Nature. Material Screening [27, 42]. Copyright (2018) American Physical Society, Copyright (2018) American Chemical Society

Currently, the research on the application of AI in traditional lithium-ion batteries has achieved substantial results [36–39]. However, the relevant research in the field of solid-state batteries is relatively scarce. In particular, for the reviews on the integration of AI and solid-state batteries [40], most of them only focus on a specific dimension of material screening or performance evaluation, lacking a comprehensive and systematic analysis of this field. This article will deeply explore the enabling effects of AI in solid-state batteries (Fig. 1) around five key directions: material screening, SOC estimation, SOH estimation, RUL prediction, and battery capacity estimation. By analyzing the current application status of AI, this article elaborates on the performance improvements, technological breakthroughs, and innovative application examples it brings to solid-state batteries. This review aims to provide a comprehensive and systematic reference for researchers, engineers, and other professionals engaged in the research and development of solid-state batteries, as well as those in the interdisciplinary field of AI and battery technology, thereby facilitating the acceleration of the commercialization process of solid-state battery technology. The structure of this review is as follows: Section 2 elaborates in detail on the application methods, typical cases, and achievements of AI in the material screening of solid-state batteries. Section 3 focuses on the application of AI in performance evaluation, covering the construction of different models and case analyses, and conducts a detailed comparison of the performance of each model. Section 4 comprehensively summarizes the main challenges faced in the integration of solid-state batteries and AI, such as issues of data quality and quantity, model adaptability and interpretability, etc., and looks ahead to the future research directions, providing clear ideas for subsequent research.

## Application of Artificial Intelligence in Material Screening

**Fig. 2:**
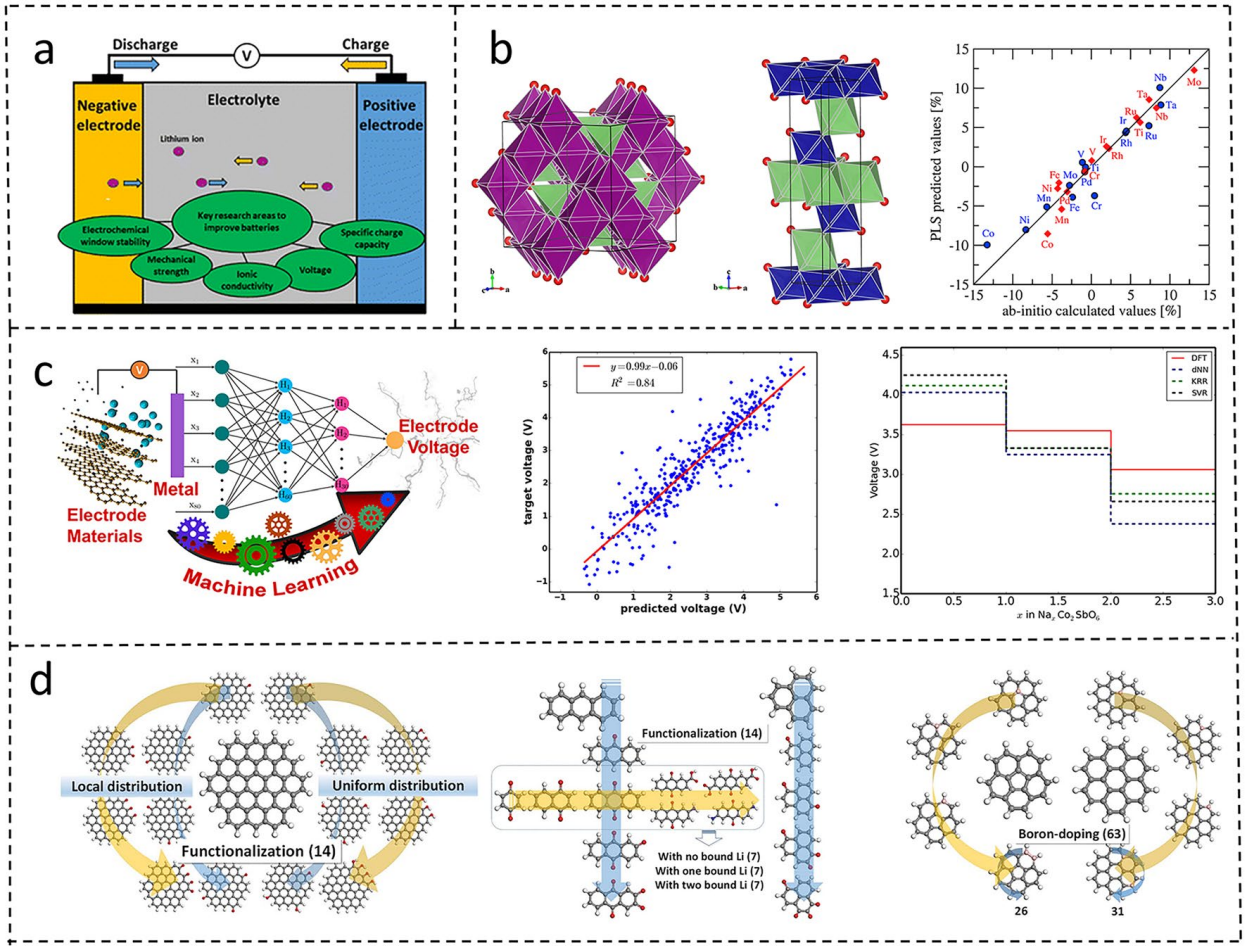
**a** Schematic of a battery cell showing the major material components (electrodes and electrolyte) and the key material properties actively under research [43]. Copyright (2023) The Royal Society of Chemistry. **b** Crystal structure diagram of spinel LiX_2_O_4_ and layered Li_X_O_2_ and cathode volume change predicted by PLS model and ab initio calculation [56]. Copyright 2017 The Chinese Ceramic Society. **c** Joshi et al.’s workflow diagram and some examples of the results [54]. Copyright 2019 American Chemical Society.** d** Molecules used to train an artificial neural network [58]. Copyright 2023The Royal Society of Chemistry

As a complex electrochemical system, a battery is mainly composed of two key components: the electrodes and the electrolyte (Fig. 2a [43]). The electrodes consist of the positive electrode and the negative electrode. When designing the electrodes, voltage and specific charge capacity play a decisive role in determining the total energy density of the battery. The electrolyte can be classified into liquid electrolytes [44–47] and SSEs [48–52]. Different types of electrolytes have different focuses on key performance parameters. For liquid electrolytes, the key parameters are the redox potential and the stability window, while for SSEs, ionic conductivity and mechanical strength need to be given priority attention. It is worth emphasizing that all the above-mentioned parameters related to battery performance are closely associated with the selected materials. In this section, we will mainly introduce the applications of ML and DL techniques in predicting material properties and material screening.

### Electrode Materials Discovery

#### Cathodes

Leveraging the comprehensive materials project (MP) database, recent studies have demonstrated the versatility of ML in accelerating the discovery of advanced battery materials. Zhou et al. [53] employed crystal graph convolutional neural networks (CGCNN) to screen nearly 13,000 inorganic compounds from the MP database, identifying over 80 promising cathode candidates with validated alignment between predicted and experimental high-voltage and high-capacity properties. Similarly, Joshi et al. [54] utilized deep neural networks (DNN) trained on MP-derived feature vectors (e.g., lattice parameters, electronic descriptors) to predict electrode voltages of metal-ion batteries within one minute, proposing 5,000 novel candidates for Na-/K-ion systems (Fig. 2c). Complementing these efforts, Sturman et al. [55] applied random forest (RF) models to analyze > 2,000 MP entries, correlating structural features with energy density to pinpoint LiNi_0.2_Mn_0.2_Co_0.2_Fe_0.2_Ti_0.2_O_2_ as an optimal high-entropy cathode, achieving enhanced stability and electrochemical performance. Collectively, these works underscore the MP database’s pivotal role in enabling diverse ML-driven workflows-from voltage prediction to multi-component material design-establishing a robust foundation for high-throughput discovery of next-generation battery materials.

Several studies integrated theoretical calculations with AI methods. Wang et al. [56] combined ab initio calculations with partial least squares (PLS) analysis to conduct research on the positive electrodes of lithium-ion batteries. Focusing on spinel LiX_2_O_4_ and layered LiXO_2_ oxides (X = various elements), they identified the radius of X^4^⁺ ions and X octahedron descriptors as key determinants of cathode volume change during deintercalation. These findings enable virtual screening and combinatorial design of low-strain cathode materials (Fig. 2b). Similarly, Sarkar et al. [57] proposed the combination of artificial neural networks (ANN) and quantum-mechanical calculations based on first-principles to predict the electrochemical potential of cathode materials and the voltage of lithium-ion batteries. Furthermore, Allam et al. [58] employed the density-functional theory-machine learning framework to devise a high-throughput screening strategy for novel molecular electrode materials. A quasi-Newton-trained ANN enabled precise redox potential prediction (Fig. 2d).

**Fig. 3:**
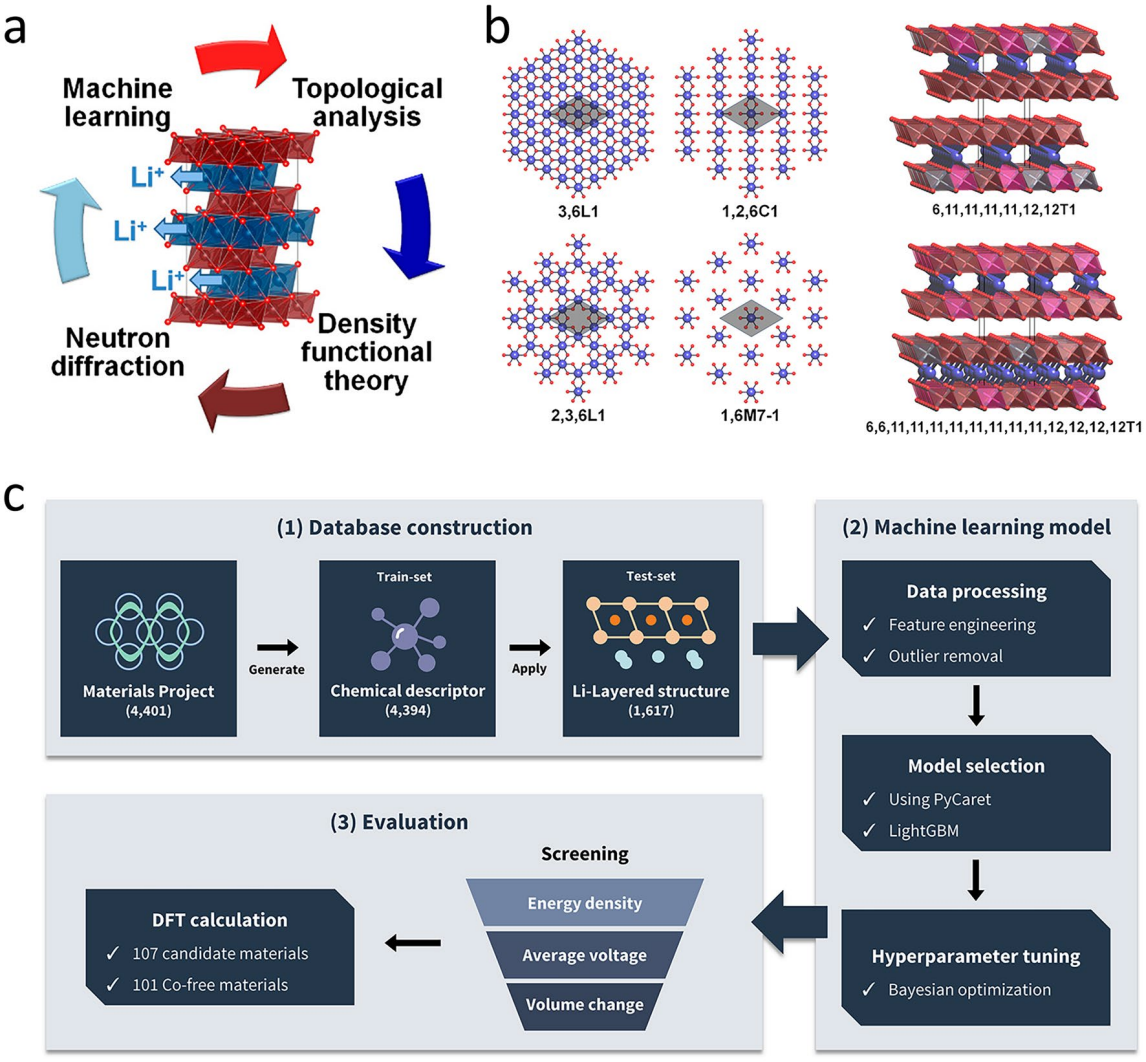
**a** Material analysis diagram combined with machine learning [60]. **b** The different values of the possible motifs of the lithium layer corresponding to the Li atom in the layer indicated by the LiO_net_2D descriptor and the two denoted configurations of the LiO_net_3D descriptors for the lithium layer 1,2,6C1 with the same motifs [60]. Copyright 2017 American Chemical Society. **c** A process flowchart constructed for predicting cathode materials using ML in combination with a database [61]. Copyright 2023, Elsevier

In addition, AI techniques have exhibited dual capabilities in advancing battery material research: facilitating high-throughput candidate screening and enabling in-depth analysis of structure–property relationships. Shandiz et al. [59] used multiple ML algorithms to analyze 339 kinds of cathode materials containing specific Li-Si-(Mn, Fe, Co)-O components. The results of data analysis clearly confirm that there is a strong correlation between the crystal structure of the cathode and other physical properties Similarly, Eremin et al. [60] combined topology/density functional theory (DFT)/ML to identify Li-layer descriptors for NCA energy balance. ML analysis shows that the topology of the Li layer and the relative configuration of Li and Al are important descriptors for the energy balance estimation of NCA, and ridge regression (RR) training gives a satisfactory level of absolute error in the prediction of configuration energy (Fig. 3a, b). Kim et al. [61] devised ML-driven strategy to screen dopants for nickel-rich cathodes. By training an ML model on 4401 material datasets, they identified 107 high-capacity candidates with minimal volume change during cycling (Fig. 3c). Notably, 101 Co-free compounds demonstrated superior chemical stability, exemplifying AI’s role in refining existing materials.

#### Anodes

**Fig. 4:**
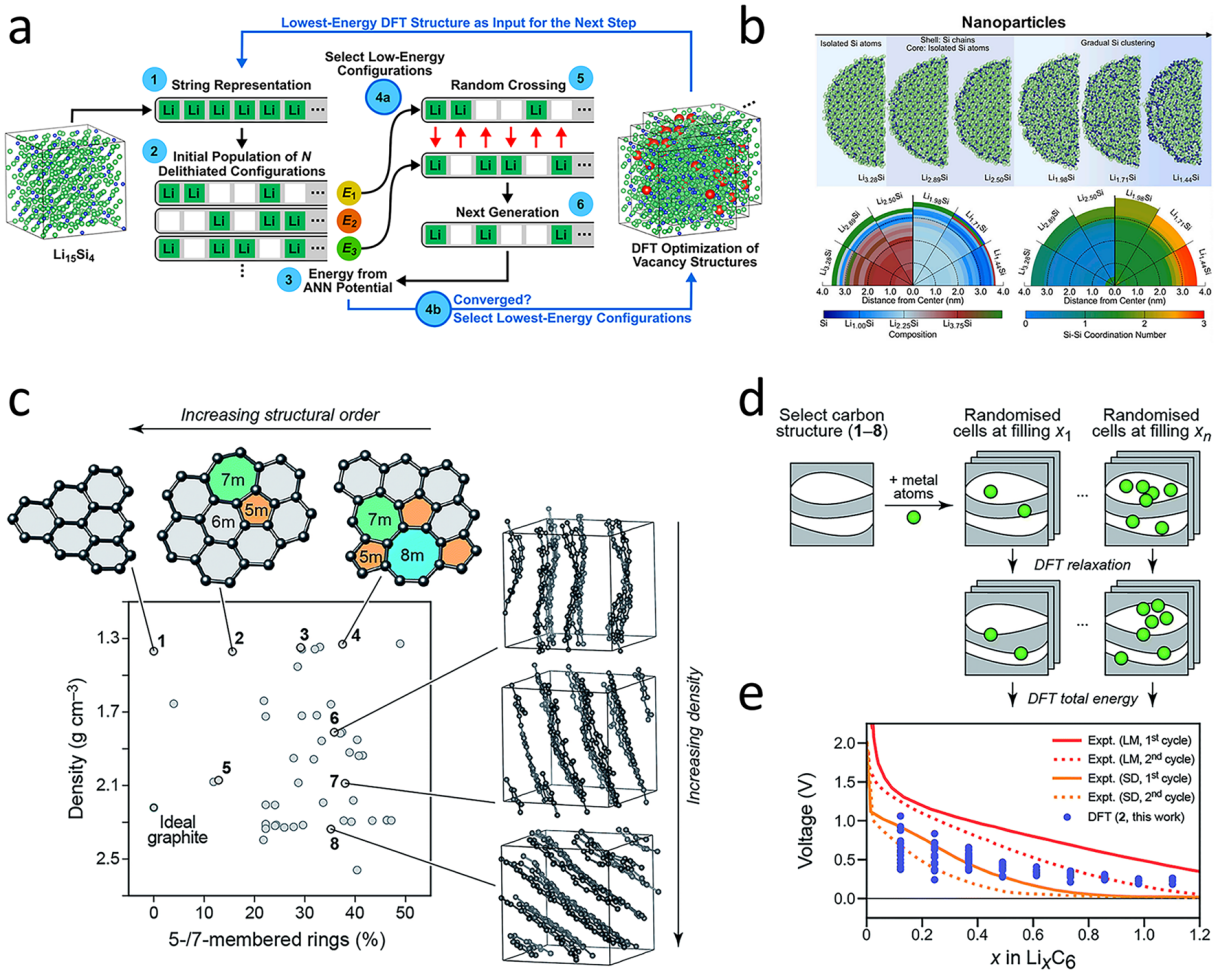
**a** Schematic of the amorphous Li_x_Si phase diagram constructed using ANN Potential assisted Genetic Algorithm (GA) [62]. Copyright 2018 American Institute of Physics. **b** During the decomposition process of a silicon nanoparticle containing 12,000 atoms, silicon atoms dissociate into clusters and chains [63]. Copyright 2019 arXiv. **c** Graphite-like structure model library is the basis of this work [65]. **d** Diagram of the protocol used to obtain the voltage-filling relationship [65]. **e** Example of resulting data [65]. Copyright 2019 Royal Society of Chemistry

Amorphous silicon-lithium alloys are widely used as anodes for high-energy density batteries. Artrith et al. [62] developed a genetic algorithm integrated with an ANN-based machine learning potential to explore amorphous silicon anodes, enabling sampling of low-energy configurations across the Li_x_Si phase space (Fig. 4a), thereby resolving the issue that the structural dimensions and sampling statistics necessary for atomic modeling of amorphous materials are difficult to achieve using first-principles methods. Complementarily, in an attempt to tackle the issue of the low rate-performance of nanostructured silicon as a high-capacity anode material for lithium-ion batteries and to gain in-depth insights into the factors governing lithium diffusion within amorphous lithium–silicon alloys, they leveraged a potential trained on 40,000 + ab initio calculations and visualized the delithiation process of lithium–silicon nanoparticles for the first time. Results revealed that silicon matrix rearrangement from isolated atoms to clusters enhanced lithium diffusion, with the highest rate occurring via cluster-to-cluster hopping. This identified silicon cluster size and aggregation concentration as key design parameters for high-rate anodes (Fig. 4b) [63].

In addition, Onat et al. [64] introduced an “Implantable Neural Network” method, extending the traditional training paradigm by incorporating pre-trained segments that can capture the unique features of different components into the overall network architecture, thus optimizing the simulation of material behaviors. This approach demonstrated superior adaptability to amorphous silicon–lithium alloys across compositions, accurately predicting diffusion coefficients, and can be conveniently applied to the modeling of more complex material systems involving two or more different elements. Similar AI-driven strategies are proving transformative for carbon-based anodes. Transitioning to graphite and non-graphitized carbon materials, due to the complex structures of disordered “hard” carbon and nanoporous carbon, conducting similar research on them faces numerous challenges. To address this, Huang et al. [65] innovatively combined the DFT with the ML method to investigate the intercalation of alkali metal (Li, Na, K) atoms in model carbon systems with different densities and degrees of disorder. By stochastically calculating voltage-filling curves for Li/Na/K intercalation in model carbons, they achieved atomic-level insights into alkali metal behaviors, advancing DFT/ML-based energy material modeling (Fig. 4c-e).

### Solid Electrolyte

#### AI-Driven Solid Electrolyte Discovery

**Fig. 5:**
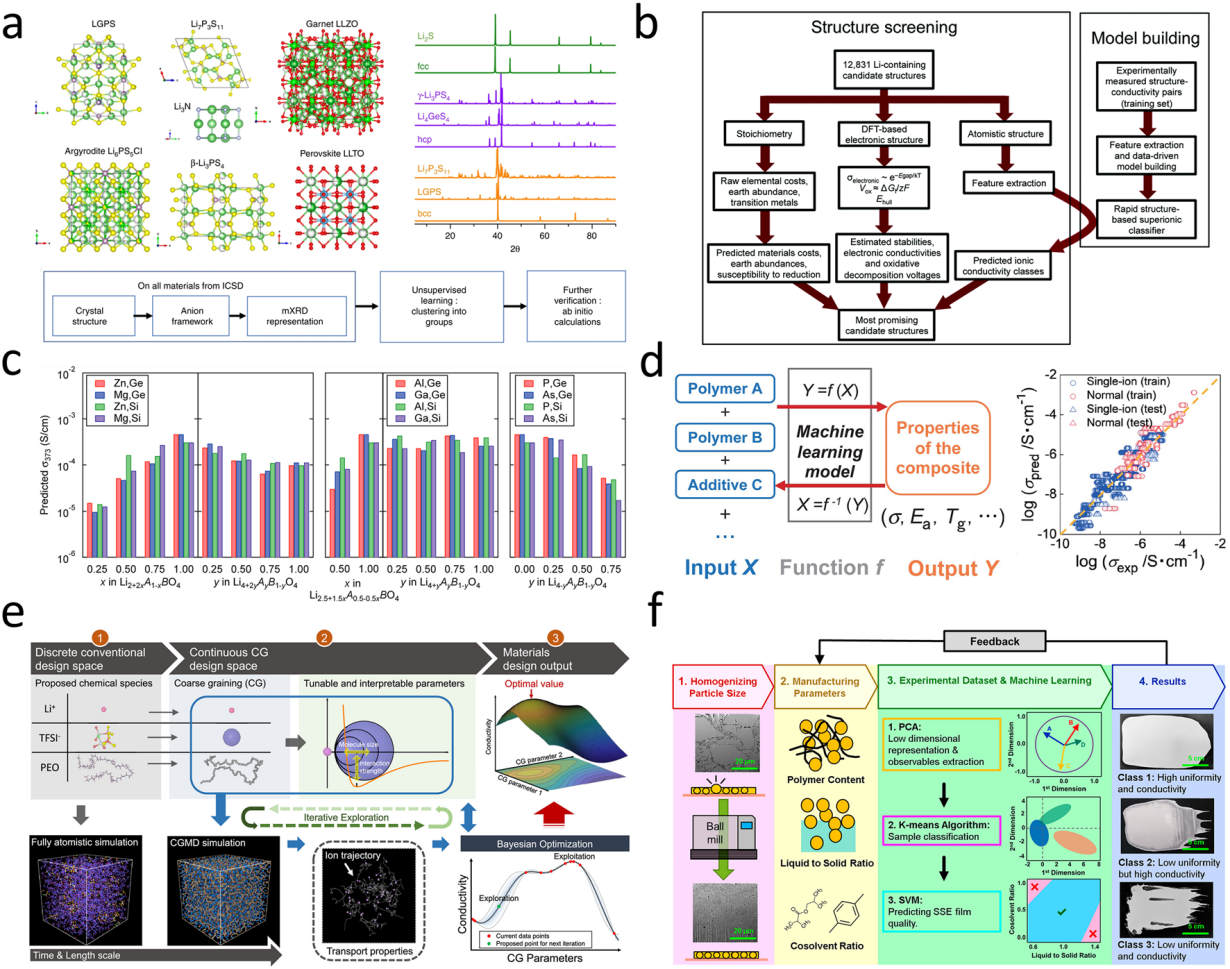
**a** Schematic diagram of screening solid lithium-ion conductors by unsupervised learning methods [67]. Copyright 2019 Springer Nature. **b** Method flow chart of building ionic conductivity model and structure screening [69]. Copyright 2017 The Royal Society of Chemistry. **c** Predicted ionic conductivity of 72 components in Li_8−c_A_a_B_b_O_4_ system at 373 K [71]. Copyright 2013 WILEY–VCH Verlag GmbH & Co. KGaA, Weinheim. **d** Machine learning-based performance prediction of solid polymer electrolytes and Comparison diagram between experimental and predicted conductivity [75]. Copyright 2019 Chemical Society of Japan. **e** Schematic diagram of coarse-grained molecular dynamics-Bayesian optimization (CGMD-BO) framework [77]. Copyright 2020 American Chemical Society. **f** Method diagram of SSE thin film evaluation with machine learning technology [78]. Copyright 2021 American Chemical Society

ML has revolutionized various fields [66], and its application in the discovery of functional materials for batteries has shown great potential. This has led to significant progress in the discovery of new materials for solid-state batteries. Zhang et al. [67] proposed a material discovery method based on unsupervised learning. This method does not require labeled data and effectively addresses the problem in which the scarcity of data hinders the progress of models in the discovery of functional materials using machine learning. Taking solid-state lithium-ion conductors as a model, the method utilized limited conductivity data to screen a candidate list of lithium-containing materials. Eventually, 16 novel fast lithium conductors were discovered (Fig. 5a). This approach was extended to Hofmann-type complexes with 2D Li⁺ channels, where weakly coordinated microenvironments were optimized via ML-guided synthesis, achieving 65% capacity retention over 500 cycles in Li||SPAN batteries [68]. Similarly, Sendek et al. [69] screened 12,831 lithium-containing solids according to the criteria of high structural and chemical stability, low electronic conductivity, and low cost, narrowing candidates to 21 high-potential SSEs (Fig. 5b). Breakthroughs in antiperovskite screening have been achieved through the identification of a geometric-kinetic descriptor (*t*/*η*), which guides the synthesis of highly conductive lithium-based nitrohalide material [70]. Fujimura et al. [71] combined the theoretical and experimental datasets and employed the SVM method to directly evaluate the ionic conductivity of Li_8−c_A_a_B_b_O_4_ LISICON. They identified several compositions with ionic conductivities higher than those of previously known LISICON (Fig. 5c). Furthermore, the integration of bond valence methods with graph neural networks enables efficient screening of 329 candidate materials, among which 28 exhibit exceptional compatibility with lithium metal [72].

In response to the challenge of screening inorganic solid-state electrolytes (ISSEs), Chen et al. proposed a ML-assisted hierarchical screening strategy. By pre-screening 20,717 lithium-containing materials, constructing a support database, and combining classification and regression models with molecular dynamics simulations, they identified three materials with high ionic conductivity at room temperature, such as Li_3_BiS_3_, and evaluated their compatibility with cathodes [73]. On the other hand, Guo et al. used a general machine learning interatomic potential (ML-IAP) to replace DFT for high-throughput calculations and identified 130 new promising solid-state electrolyte materials. Through parameterizing the screening criteria and implementing a hierarchical evaluation process, they revealed the crucial influence of characteristics such as the maximum packing efficiency on Li^+^ conduction [74].

#### Composite Electrolyte Design

Hatakeyama-Sato et al. [75] utilized machine learning to conduct an analysis of a database that encompasses 240 lithium-ion conducting solid polymer electrolytes. Subsequently, polyglycol ether derivatives with high electrical conductivity were synthesized. In addition, based on the constructed database, single-ion conducting polymers with de novo design were screened out from over 15,000 candidates (Fig. 5d). Machine learning-guided screening of dual-doped LLZO fillers yields Zn-Ti-based PVDF-HFP composite electrolytes, which exhibit an ionic conductivity of 8.7 × 10⁻^4^ S cm⁻^1^ and a 4.8 V electrochemical stability window, significantly enhancing ASSB performance [76]. Wang et al. [77] constructed a continuous high-dimensional design space through the coarse-graining of chemical substances and employed the Bayesian optimization algorithm to explore this space. As a result, a comprehensive description of the relationship between lithium conductivity and molecular properties was obtained, providing guidance for improving the composition of electrolytes (Fig. 5e). Meng et al. [78] leveraged ML techniques to evaluate SSE films (Fig. 5f). Eventually, with the assistance of ML, a LiNi_0.8_Co_0.1_Mn_0.1_O_2_||Li_6_PS_5_Cl||LiIn battery constructed using a 40-μm-thick high-quality SSE film successfully completed 100 cycles. This achievement not only highlights the importance of considering both uniformity and ionic conductivity during the fabrication of SSE films but also fully demonstrates the remarkable advantage of ML in guiding experiments to determine the optimal manufacturing parameters.

#### Ion Transport Mechanisms

The development of solid-state batteries necessitates overcoming critical challenges in defect structure and transport mechanisms. Addressing metallic anode penetration, recent research reveals that mixed ionic-electronic conduction in solid electrolytes induces lithium deposition within micrometer-scale voids, proposing dendrite suppression strategies through optimized control of cell voltage and applied current density [79]. In the optimization of oxide-based electrolytes, defect chemistry studies on garnet-type Li_7_La_3_Zr_2_O_12_ elucidate the influence of temperature and oxygen partial pressure (PO_2_) on ionic conductivity via AC impedance and DC polarization techniques. The construction of defect equilibrium diagrams provides theoretical guidance for designing high-conductivity materials (2 mS cm^−1^ at room temperature) [80]. Additionally, oxygen vacancy regulation in lithium zirconate demonstrates that Fe(II) doping enhances Li⁺ conductivity to 3.3 mS cm^−1^ at 300 °C-an order of magnitude higher than undoped materials-revealing a direct correlation between oxygen vacancy concentration and ion transport efficiency [81]. These advancements systematically advance solid electrolyte systems through failure mechanism analysis, defect engineering, and ion transport optimization. Meanwhile, these provide experimental data support for future artificial intelligence-based analyses.

**Fig. 6:**
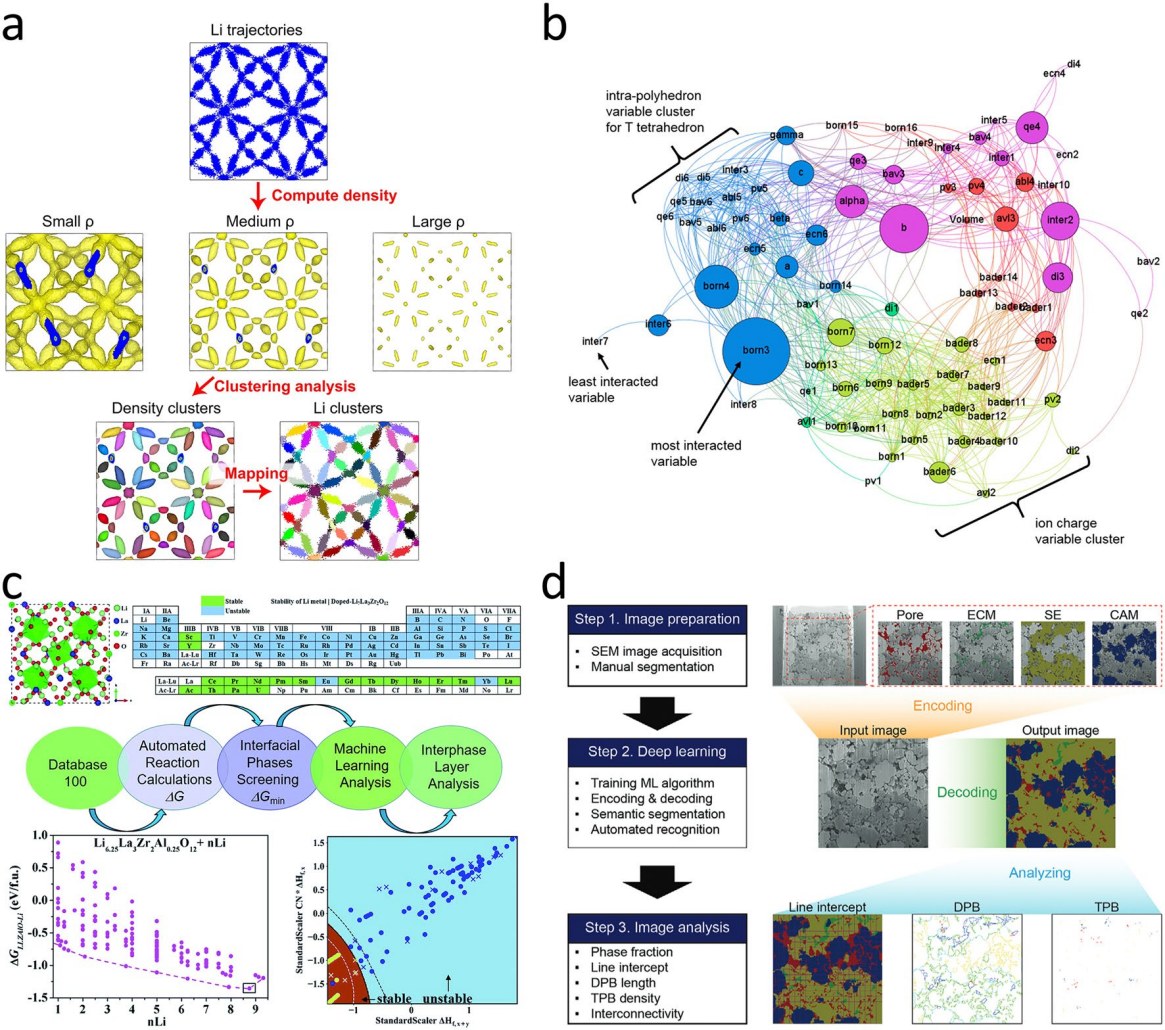
**a** 400 K simulation of the cubic LLZO as shown in the clustering scheme [83]. Copyright 2017 Springer Nature Limited. **b** According to Pearson’s product moment correlation coefficient R, a graph theory-based network constructed by variable interaction effects [84]. Copyright 2015 American Chemical Society. **c** Overall schematic of selecting the right dopant in LLZO [91]. Copyright 2019 The Royal Society of Chemistry. **d** A strategy for assisting microstructure analysis by semantic segmentation in all-solid-state batteries [93]. Copyright 2025 John Wiley & Sons

DL interatomic potential simulations have elucidated the “soft” lithium-hopping behavior induced by structural disorder in Li_3_PS_4_, establishing a machine learning-derived structural fingerprint (“softness”) to quantify ion migration dynamics [82]. This complements Chen et al.’s [83] spatiotemporal analysis of Li⁺ diffusion in LLZO using density-based trajectory clustering, which uncovered uncorrelated Poisson-like migration in cubic phases (Fig. 6a). Furthermore, inspired by the fact that the tavorite structure can provide a fast lithium-ion insertion rate, Jalem et al. [84] constructed a prediction model for the lithium migration energy (ME) based on the crystal structure. With the help of this model, the researchers identified a series of candidate components with low lithium migration energy, such as LiGaPO_4_F and LiGdPO_4_F (Fig. 6b).

#### Interface Engineering

The development of high-performance solid-state sodium-metal batteries (SSSMBs) hinges on resolving interfacial instability and dendrite propagation challenges. A biphasic Na_3_Zr_2_Si_2_PO_12_/Na_3_PO_4_ electrolyte demonstrates enhanced ionic conductivity (6.2 × 10⁻^4^ S cm⁻^1^) and self-forming Na_3_P/Na_2_O interphases, enabling 550-cycle stability (93% capacity retention) in full cells through uniform Na⁺ flux distribution and suppressed interfacial reaction [85]. Similarly, a plastic monolithic mixed-conducting interlayer (PMMCI) of Li-Naph(s) achieves dual ionic (4.38 × 10⁻^3^ S cm⁻^1^) and electronic (1.01 × 10⁻^3^ S cm⁻^1^) conduction, reducing Li/garnet interfacial resistance and enabling 500-h dendrite-free cycling at 1 mA cm⁻^2^ [86]. For NASICON-based systems, a comprehensive review identifies interfacial challenges (e.g., high resistance, dendrites) and categorizes mitigation strategies, emphasizing mechanical/chemical stabilization mechanisms to guide future ASSSMB designs [87]. Innovative interface engineering via Fe-valence-graded fluorinated interphases in Na_3_Zr_2_Si_2_PO₁^2^-based cells reduces interfacial resistance by 20 × and achieves 1.9 mA cm⁻^2^ critical current density, enabling 96% capacity retention over 120 cycles [88]. Finally, a Pb/C interlayer strategy achieves perfect Na wetting (0° contact angle) at 120 °C and ultralow interfacial resistance (1.5 Ω cm^2^), facilitating 1800-h stable cycling in symmetrical cells and optimized N/P ratio-dependent full-cell performance [89]. These studies collectively advance interfacial design paradigms through phase modulation, interlayer engineering, and dynamic interphase formation, addressing critical barriers in SSSMB commercialization. Meanwhile, these provide experimental data support for future artificial intelligence-based analyses.

When shifting to the lithium-metal system, the problem of dendrite growth remains equally severe. However, the solutions are more reliant on the collaboration between multi-scale simulations and ML. At the Li/SSE interfaces, parallel progress in electrode-electrolyte interface optimization has been facilitated by atomic-scale simulations. ML potentials reveal cobalt segregation at grain boundaries in LLZO/LCO interfaces, clarifying its inhibitory effects on lithium-ion transport and providing atomic-level insights for interface modification [90]. For lithium-metal anodes, Liu et al. [91] employed SVM/KRR models to identify dopants (e.g., Sc^3^⁺, Ca^2^⁺) that stabilize interfaces via SEI formation, Through ML analysis, it was discovered that the strength of the M–O bond plays a decisive role in the interface stability of cation-doped LLZOM. This research achievement provides valuable theoretical basis and practical guidance for experimental researchers to screen appropriate dopants in LLZO, thus stabilizing the lithium-metal anode in solid-state batteries (Fig. 6c). Similarly, Wang et al. [92] constructed a stable and highly ion-conductive molten salt interface (MSI). Through this MSI, the interfacial contact was improved, and interfacial reactions and thermal runaway were inhibited. The construction of the MSI provides a direct and effective traditional experimental approach to address the interface issues between the lithium anode and the solid-state electrolyte. In addition, advances in microstructure characterization are exemplified by semantic segmentation of electron micrographs, enabling automated quantification of porosity and phase distribution in composite cathodes, thereby correlating microstructural parameters with battery performance and providing data support and research directions for interface engineering (Fig. 6d) [93].

### Key Mechanisms for Screening Materials

In solid-state battery research, material exploration and discovery are crucial and can be achieved through three interconnected phases. The first phase is constructing targeted material databases. Clearly, define research objectives. For solid-state electrolytes, focus on parameters like ionic conductivity, electrochemical stability, electrode interfacial compatibility, mechanical robustness against dendrite formation, and wide-temperature adaptability. Generate high-quality data through experimental characterization (e.g., XRD for crystal structure and EIS for interfacial resistance) and literature mining on platforms such as Web of Science and Scopus. This creates a database that maps structure-property-processing correlations, supporting novel material design.

The second phase is using ML and DL for material screening. First, extract relevant features from raw data, such as lattice parameters and bond lengths for crystalline materials, which affect properties like ionic conductivity. Then, choose appropriate models according to task complexity. ML models like decision trees and random forests are good for simple tasks (e.g., classifying materials by conductivity), while DL models like CNNs are better for complex relationships (e.g., predicting interfacial stability from HRTEM images).

The third phase involves key mechanisms that accelerate material discovery. ML and DL models pre-screen a large number of materials in databases, reducing the number of experimental tests, saving time, and cutting costs. They also accelerate design cycles by accurately predicting material properties, allowing for efficient and iterative material design. Additionally, data mining with these models can uncover hidden patterns in databases, leading to the discovery of new materials and design principles for solid-state batteries. Table 1 shows a comparison highlighting the accuracy, model complexity, computational cost, practical feasibility, advantages and limitations of various AI techniques for different battery components.

**Table 1:** Comparison of various AI techniques for different battery components

Method	Material types	Accuracy	Complexity	Computational cost	Feasibility	Advantages	Limitations	References
CGCNN	Cathode	–	Complex graph structure modeling	time-consuming for large datasets	Feasible when there is sufficient data	Automatically extracts crystal features and has a good perception of the global structure	Sensitive to data, high hardware requirements, and difficult to interpret	[53]
DNN	Cathode	–	Multiple layers of neurons with a large number of parameters	Requires GPU clusters for training, high cost	Feasible with large datasets and strong computing power	Automatically extracts features and has strong fitting ability	Prone to overfitting, and the training process is time-consuming	[54]
RF	Cathode	–	Composed of multiple decision trees, with adjustable parameters	Fast parallel computing, low cost	Strong universality, requiring less data preprocessing	Resistant to overfitting	Weak in fitting complex relationships	[55]
ANN	Cathode Anode	–	Flexible structure (ranging from simple to complex)	Fluctuates depending on the scale, moderately controllable	Applicable to small and medium-sized datasets	Capable of handling nonlinear problems, with an adjustable structure	Prone to getting trapped in local optimal solutions, and a large amount of data is required for complex tasks	[57] [62]
SVM	Solid electrolyte	–	The choice of kernel function affects the complexity	Training on large datasets is slow, and has a high memory occupation	It is feasible when dealing with small to medium-sized datasets and with a moderate number of feature dimensions	It has obvious advantages in classifying high-dimensional data and has strong generalization ability	It is inefficient for large-scale data, sensitive to parameters, and the selection of the kernel function depends on experience	[71]
GNN	Solid electrolyte	–	It is necessary to construct a graph structure (node/edge features)	Graph data processing is complex, and there is a high demand for GPU acceleration	It has strong applicability to tasks related to material structure, and the data need to be structured	It can capture the topological relationships of material structures and conduct end-to-end learning	It has high requirements for data structuring, large computational overhead, and poor interpretability	[72]
gradient boosting	Solid electrolyte	R^2^ = 0.81	It constructs an ensemble of trees through multiple rounds of iteration	Single-threaded training is slow, and distributed computing is required for large datasets	It has strong adaptability to multiple scenarios and does not require complex feature engineering	It has high-precision prediction and can handle nonlinearity and feature interactions	It is prone to overfitting, the training process is time-consuming, and parameter tuning is cumbersome	[76]
K-means	Solid electrolyte	–	Only the number of clusters K needs to be specified as a hyperparameter, which is simple and intuitive	It is sensitive to the initial clustering centers and requires multiple random initializations	It can quickly partition the data, with a low computational complexity	It is lightweight and interpretable, and is suitable for pre-exploration of data	The value of K needs to be preset, the results are unstable, and it has poor performance on non-convex distributed data	[77]
KRR	Solid electrolyte	–	It depends on the kernel function (such as RBF), and the complexity of tuning is moderate	Training requires matrix inversion, and there is great memory pressure for large datasets	It has high accuracy in small sample regression tasks and strong anti-noise ability	It has excellent nonlinear fitting ability and has no risk of overfitting (due to regularization)	The kernel parameters are difficult to tune, and the computational complexity is O(n^3^) (when not optimized)	[78]

## Application of Artificial Intelligence in Performance Evaluation

The complex nonlinear interdependencies in BMS pose significant challenges for performance assessment, particularly in emerging solid-state batteries. By comparison, solid-state batteries face challenges such as the formation of interfacial resistance between the solid electrolyte and electrodes, as well as dendrite growth within the solid matrix. While machine learning has demonstrated transformative potential in traditional lithium-ion BMS through SOC/SOH estimation, its application to solid-state systems remains nascent. The achievements of ML and DL in the application of traditional lithium batteries have laid a foundation for solid-state battery research [94, 95]. Therefore, the development of accurate performance prediction models specifically for solid-state batteries is of greater importance for their successful commercialization. This endeavor could enable the construction of high-precision solid-state battery performance assessment models, enhance intelligent management levels, and ultimately guide the future development direction of battery technology.

### State-of-Charge Estimation

**Fig. 7:**
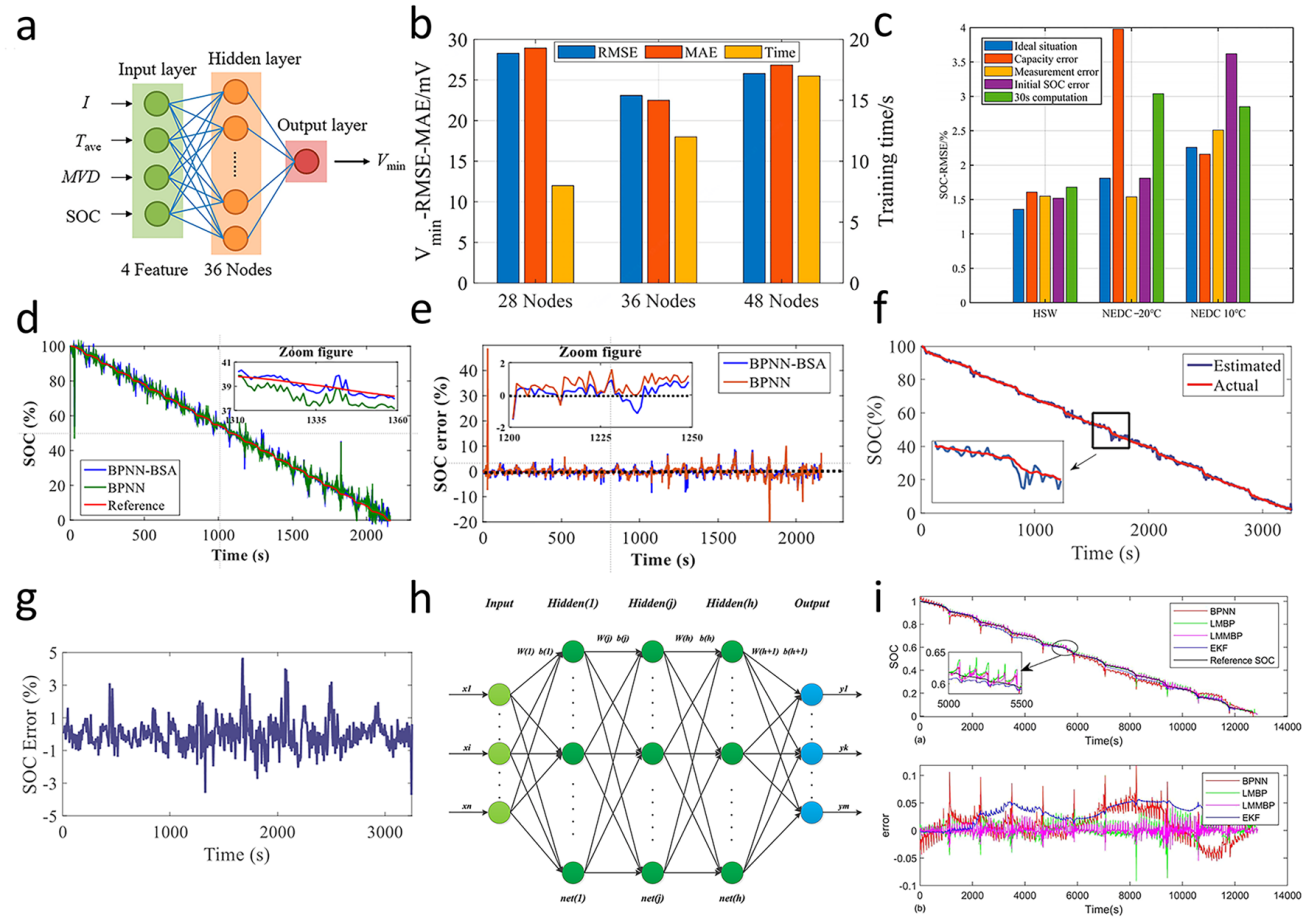
**a** Model structure of backpropagation neural network (BPNN) [84]. **b** Model training time and test set voltage RMSE under different hidden layer node numbers [98]. **c** SOC estimation results under ideal and different error conditions [98]. Copyright (2023) MDPI. **d**, **e** SOC estimation results and SOC error for FUDS cycle 0 °C [99]. Copyright (2018) IEEE. **f**, **g** SOC estimation results and SOC errors based on PSO under FUDS cycle [100]. Copyright (2017) AIP Publishing LLC. **h** Topology structure of multiple-hidden-layer BPNN [101]. **i** SOC estimation results and errors under the NEDC of BPNN, LMBP, LMMBP and EK [101]. Copyright (2021) Elsevier

Leveraging real-time monitoring and in-depth analysis of battery operation data, AI constructs precise prediction models to achieve accurate SOC tracking. As potent data analysis tools, ML and DL have yielded extensive research outcomes in the SOC estimation of lithium-ion batteries [96]. The backpropagation neural network (BPNN), a type of multilayer feedforward neural network, composed of an input layer, hidden layers, and an output layer. It is trained based on the error backpropagation algorithm. Through forward propagation, it calculates the output, compares the output with the true value to obtain the error, and then backpropagates the error to adjust the connection weights of each layer, thus continuously optimizing the network performance. The model structure is shown in Fig. 7a. It is widely used in the estimation of the SOC of lithium-ion batteries. Huang et al. [97] developed a BPNN for estimating the SOC of batteries. Taking the battery voltage and current as inputs, the real-time capacity was calculated to estimate the SOC. However, the BPNN has some problems, such as slow convergence speed and being prone to falling into local minima. To further enhance the accuracy of BPNN-based SOC estimation and leverage real-time correction capabilities, Zhang et al. [98] combined a backpropagation neural network with an extended Kalman filter, where the EKF algorithm can correct the SOC with voltage error information. At the same time, they trained a single-hidden-layer BPNN model with 28, 36, and 48 nodes, respectively, compare the simulation errors generated by the test set and training time under different node numbers, and obtain an optimal number of hidden layer nodes (Fig. 7b). The RMSE was reduced to 3.98% at −20 °C NEDC, 3.62% at 10 °C NEDC, and 1.68% at 35 °C HSW (Fig. 7c).

Given BPNN’s vulnerability to local minima during training, Aini et al. [99] employed the backtracking search algorithm (BSA), a depth-first search-based technique, to enhance the performance of BPNN (Fig. 7d, e). BSA commences from an initial state, incrementally constructing solutions while systematically verifying constraint conditions at each iteration. When a partial solution violates constraints or reaches a dead end, the algorithm backtracks to the previous state to explore alternative paths. This iterative process exhaustively searches the problem space until a valid solution is found or all possibilities are exhausted, enhancing the accuracy and robustness of the BPNN model. To address the challenges of data complexity and improve model generalization. Hossain et al. [100] adopted the principal component analysis (PCA) and particle swarm optimization (PSO) to improve the BPNN and obtained better robustness. The range SOC error of this model for the Federal Urban Driving Schedule (FUDS) was between 3.7% and 4.7% (Fig. 7f, g). PCA, a statistical technique for data analysis and dimensionality reduction, transforms correlated variables into uncorrelated principal components. By maximizing variance projection, PCA condenses data while preserving key information, aiding visualization and analysis. Complementarily, PSO draws inspiration from bird flocking and fish schooling behaviors and optimizes solutions through a group of particles adjusting positions based on personal and global bests. Together, PCA and PSO synergistically refine the BPNN’s performance for battery SOC prediction. Aiming to exploit deeper network architectures for capturing intricate patterns in battery data, Zheng et al. [101] proposed a multi-hidden-layer BP neural network (LMMBP) trained based on the Levenberg-Marquardt (L-M) algorithm. Its structure is shown in Fig. 7h, and the error is shown in Fig. 7i. The output results of the BPNN were optimized by increasing the number of hidden layers. The RMSE was reduced to 0.5%.

**Fig. 8:**
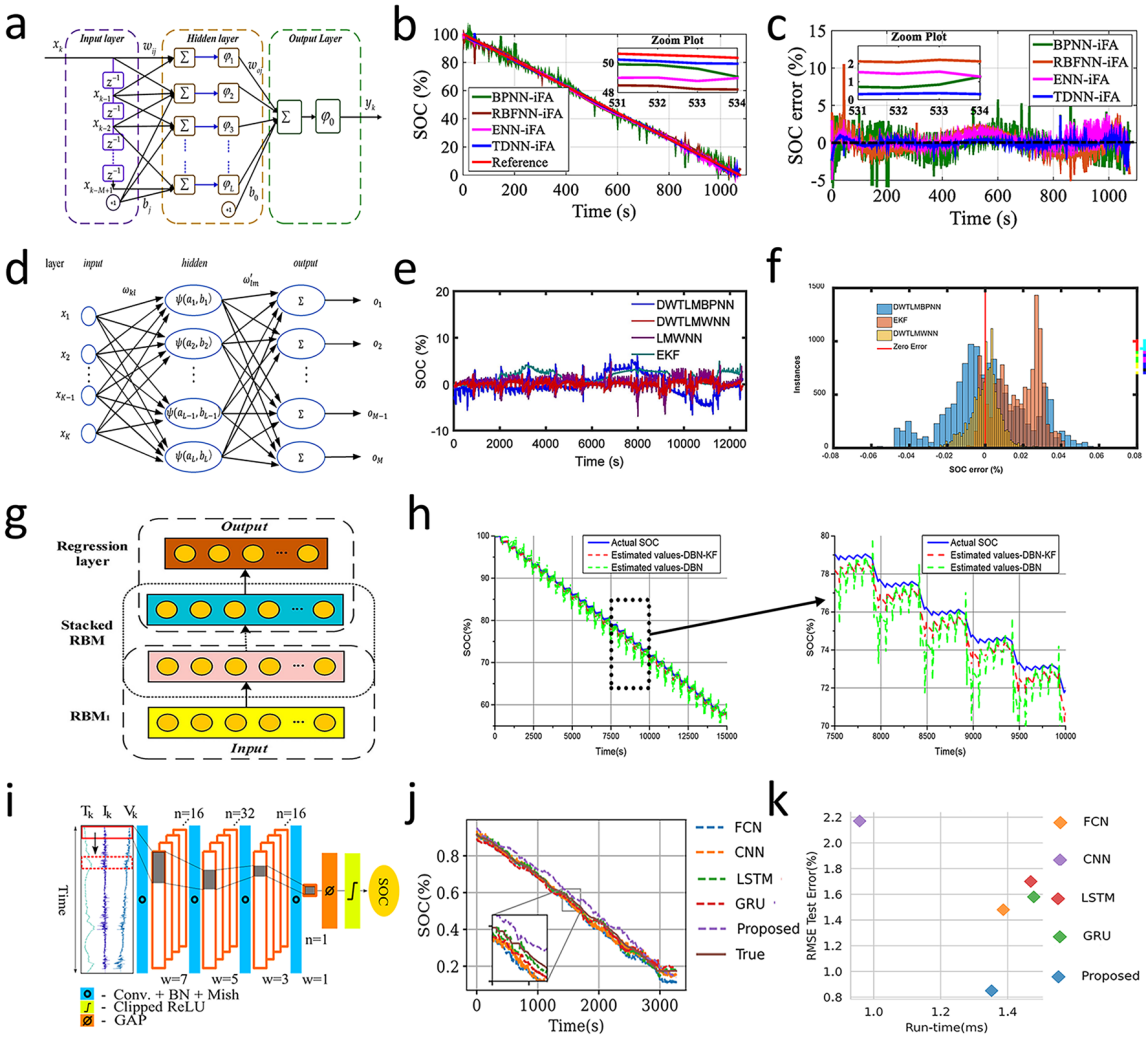
**a** Model structure of TDNN [102]. **b, c** SOC estimation results and SOC errors under SDT [102]. Copyright (2020) MDPI. **d** Structure of a three-layer WNN [103]. **e, f** SOC estimation and SOC estimation error histogram using DWTLMBPNN, EKF, and DWTLMWNN [103]. Copyright (2018) MDPI. **g** DBN model structure [104]. **h** Comparison results under DST test [104]. Copyright (2019) Elsevier. **i** FCN model structure [105]. **j, k** SOC estimation under US06 at 0 °C and RMSE error values of each comparison model [105]. Copyright (2020) IEEE

In addition, the time delay neural network (TDNN) (Fig. 8a) is a supervised ML algorithm with a special neuron connection, which can handle the delay information in time series data. Given the sensitivity of TDNN to hyperparameters for time series processing, Hossain et al. [102] optimized the TDNN algorithm through the improved firefly algorithm (iFA) to determine the optimal input time delay (UTD) and the number of hidden neurons (HNs). The results showed that the iFA-based TDNN obtained accurate SOC estimation results (Fig. 8b, c), and the RMSE was lower than 1%.

Furthermore, the wavelet neural network (WNN) integrates wavelet analysis with traditional neural networks. Using wavelet functions as activation or basis functions instead of sigmoid-like ones, WNN has enhanced local approximation and multi-resolution analysis capabilities. Wavelet basis functions capture fine and transient data features, helping WNN outperform traditional networks in handling non-stationary and complex datasets (Fig. 8d). Cui et al. [103] combined the DWT with an L-M trained adaptive WNN to create a DWTLMBPNN for lithium-ion battery SOC estimation, achieving an MAE of 0.59% and a maximum error of 3.13% (Fig. 8e, f). Liu et al. [104] combined the DBN (Fig. 8g) and KF to create a hybrid model for dynamic lithium-ion battery SOC estimation. The DBN extracts parameter–SOC relationships, and the KF reduces measurement noise. The estimation results of DBN-KF are shown in Fig. 8h, and the maximum average estimation error is less than 2.2%. Advancing from traditional CNN designs, fully convolutional architectures offer improved efficiency for sequential data tasks. Hannan et al. [105] proposed a fully convolutional network (FCN) for SOC estimation. Based on traditional CNN designs, the FCN (Fig. 8i) has four temporal convolutions and converts end-layer fully connected layers to convolutional ones for better sequential data processing. From the comparison of the prediction results of this model with those of other common models (Fig. 8j, k), it can be seen that this model has a better effect, with a RMSE of 0.85% at room temperature. Table 2 shows a comparison of the different SOC estimation method.

**Table 2:** Comparison of the different SOC estimation method

Method	Import	Prediction results	References
BPNN	V, I	Error = 0.10%—0.50%	[97]
BPNN-EKF	V, I, T	RMSE = 3.98%, -20 °C (NEDC) RMSE = 3.62%, 10 °C (NEDC) RMSE = 1.68%, 35 °C (HSW)	[98]
BPNN-BSA	V, I, T	RMSE = 0.91%, 25 °C (FUDS) RMSE = 0.57%, 45 °C (FUDS)	[99]
BPNN (PCA + PSO)	V, I, T, dv, d^2^v, di, d^2^i $\int v$,$\int i$	RMSE = 0.58% (BJDST) RMSE = 0.72% (FUDS) RMSE = 0.47% (US06)	[100]
LMMBP	V, I, T	MAE = 3.5% (UKBC)	[101]
TDNN	V, I, T	RMSE = 0.5844% (SDT) RMSE = 0.8512% (HPPC)	[102]
DWTLMWNN	V, I, T	MAE = 0.59% (NEDC) Maximum Error = 3.13%(NEDC)	[103]
DBN-KF	V, I, T	MAE < 2.2%	[104]
FCN	V, I, T	RMSE = 0.85% 25 °C MAE = 0.70% 25 °C	[105]

### State-of-Health Estimation

SOH estimation, a critical parameter in BMS, plays a pivotal role in optimizing performance, ensuring safety, and extending battery lifespan. By leveraging deep learning to analyze a battery’s full life cycle data, this approach enables insights into the evolution of its internal health and facilitates early warning mechanisms [106, 107]. In recent years, ML methods have achieved remarkable progress in the field of battery SOH estimation. Many studies have been dedicated to developing efficient and accurate SOH estimation algorithms to cope with various challenges in practical applications.

**Fig. 9:**
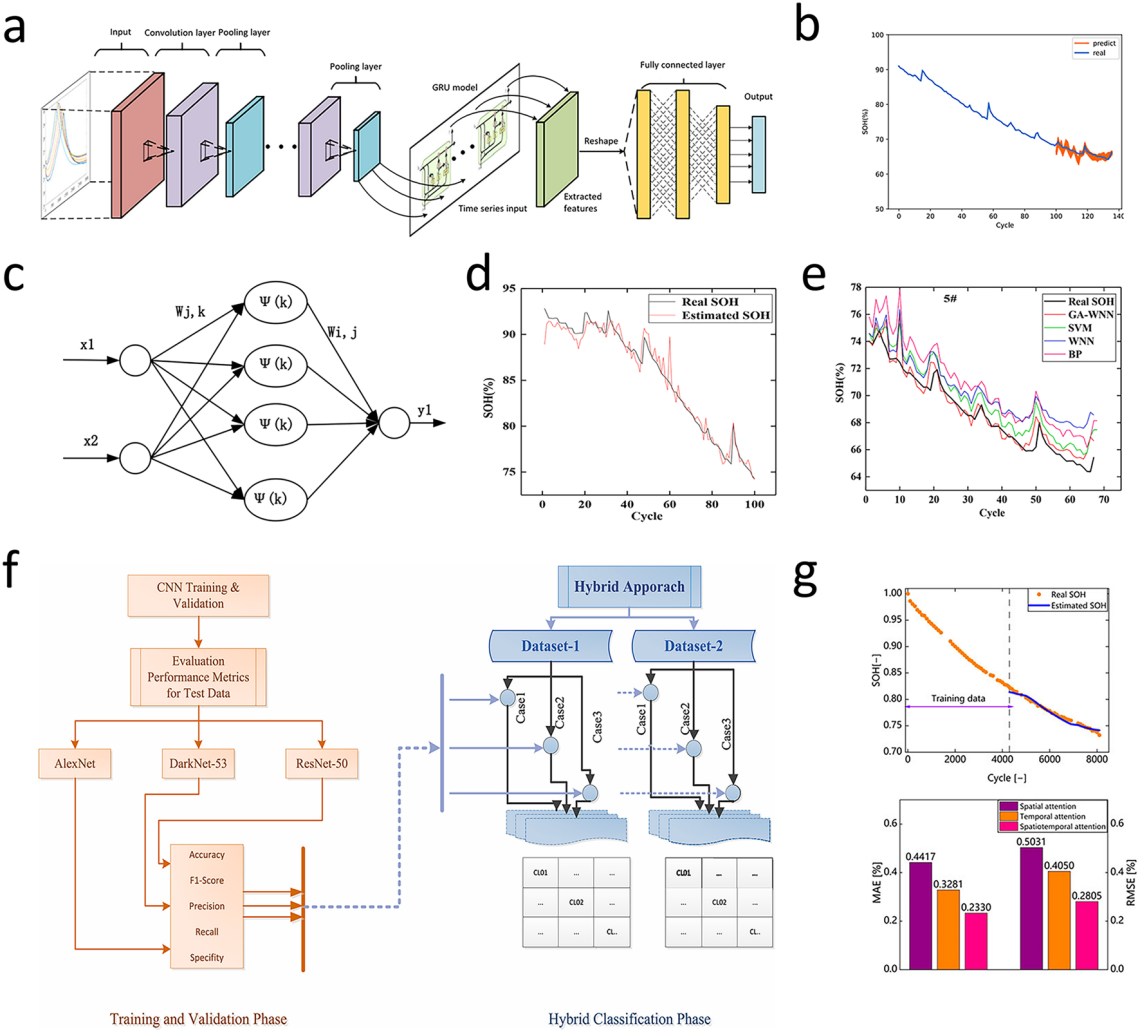
**a** CNN-GRU model structure [110]. **b** The first 100 cycles of the battery are used for training, and the last 36 cycles are tested. Predicted battery SOH [110]. Copyright (2022) Elsevier. **c** WNN model structure [112]. **d** Training effect of GA-WNN model [112]. **e** SOH estimation results based on different methods [112]. Copyright (2021) Elsevier. **f** Flowchart of hybrid decision method based on DL [113]. Copyright (2024) Elsevier. **g** Estimation result with temporal attention and MAE analysis [106, 114]. Copyright (2023) Frontiers Media

Liu et al. [108] successfully applied ELM to SOH prediction. By mapping the implicit dependence between voltage variance and health state in time intervals, it shows higher prediction accuracy and faster computation speed with RMSE less than 0.5% compared to traditional artificial neural networks. Li et al. [109] extracted four important features by analyzing the partial incremental capacity and combined Gaussian process regression with nonlinear regression to predict the battery’s SOH. The results showed that this method can accurately predict the health status of the battery. Zheng et al. [110] combined the GRU with the CNN and proposed a GRU-based SOH estimation method (CNN-GRU). The structure of this model is shown in Fig. 9a. Compared with other traditional methods, this method can achieve high-precision SOC estimation (Fig. 9b) without the need to manually construct feature information, and the MAE and mean absolute percentage error (MAPE) can reach 0.901% and 1.359%, respectively. The CNN-LSTM architecture combines CNN’s spatial feature extraction and LSTM’s time series analysis capabilities. LSTMs, a specialized RNN variant, can handle sequential data and avoid the vanishing gradient problem, with memory cells and gating mechanisms for capturing long-term dependencies. Building upon CNN-LSTM, Zhang et al. [111] introduced an advanced hybrid model, the attention-augmented CNN-LSTM (AACNN-LSTM), for battery SOH estimation. Rigorous comparative experiments across diverse training cycles demonstrated that the AACNN-LSTM architecture outperforms baseline methods, showcasing robust SOH prediction accuracy under varied operational conditions.

Despite the success of CNN-GRU, attention mechanisms further enhance model focus on critical battery health indicators. Chang et al. [112] proposed a GA-WNN-based SOH estimation method using incremental capacity features (Fig. 9c). They extracted important features related to battery health through the IC curve and then used the WNN model optimized by the genetic algorithm to estimate the SOH. Comparative experiments with traditional algorithms (Fig. 9d, e) showed that this method could keep SOH estimation error within 3%. Previous studies mainly focused on single-battery health evaluation. Multi-battery prediction, however, needs specialized strategies to handle heterogeneity. Yamacli [113] proposed a data-driven and DL-based hybrid method for predicting the health of series-connected lithium-ion batteries (Fig. 9f). The results demonstrated that the average accuracy rate of this method is 98.3%, and it can be implemented not only in online systems but also in offline systems. Facing the challenge of feature relevance variability in time and space, Zou et al. [114] used bidirectional long short-term memory (Bi-LSTM) as the core and used differential thermal voltammetry analysis for feature extraction. More importantly, the attention mechanism is incorporated into the temporal and spatial dimensions to make the model focus on the key information in the features, thus effectively improving the model performance. The results showed that RMSE and MAE were about 0.4% and 0.3%, respectively (Fig. 9g). Table 3 shows a comparison of the different SOH estimation method.

**Table 3:** Comparison of the different SOH estimation method

Method	Import	Prediction results	References
ELM	V, I, T	RMSE < 0.5%	[108]
Gaussian Process Regression	V, I, T	RMSE = 0.46% MAE = 0.31%	[109]
CNN-GRU	V, I, T	MAE = 0.901% MAPE = 1.359%	[110]
AACNN-LSTM	V, I, T	MAE = 0.63% RMSE = 0.97%	[111]
GA-WNN	V, I, T	Maximum MAE = 1.81% Maximum MAPE = 2.98%	[112]
Hybrid method	V, I, T	Maximum MSE = 0.3833% Maximum RMSE = 0.6191% Maximum MAE = 0.2058% Maximum MAPE = 4.1875%	[113]
Bi-LSTM	V, T, R	RMSE = 0.4% MAE = 0.3%	[114]

### Remaining Using Life Prediction

RUL prediction is crucial for battery management as it optimizes utilization, ensures safety, and enhances performance, and accurate RUL prediction has emerged as an essential research focus [115]. In recent years, ML and DL algorithms have achieved numerous research results in the prediction of the RUL of lithium-ion batteries, which are of great reference value for the RUL prediction of solid-state batteries. Andrioaia et al. [116] compared support vector machine for regression (SVMR), multiple linear regression (MLR), and RF to estimate battery RUL. The SVMR demonstrated relatively superior performance, with a MAE of 1.02% and a RMSE of 7.14%.

**Fig. 10:**
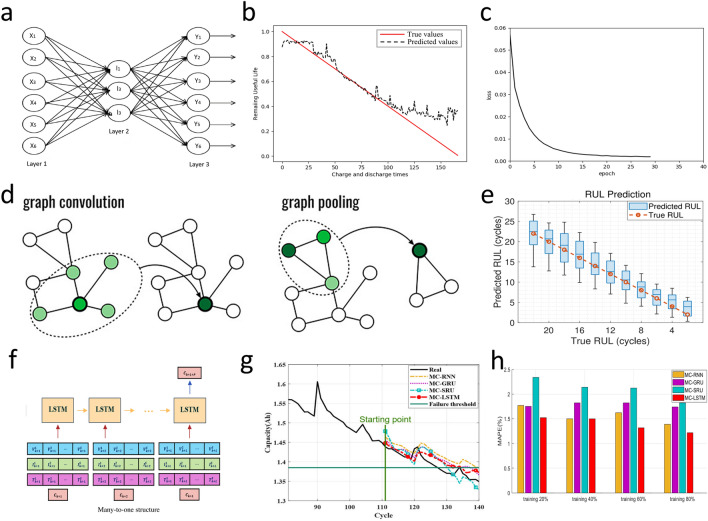
**a** Autoencoders model structure [117]. **b** Results of battery prediction using Autoencoder and DNN model (ADNN) [117]. **c** The relationship between training amount and loss function [117]. Copyright (2021) Elsevier. **d** GCN model structure [118]. Copyright (2019) American Chemical Society. **e** Box plot of RUL prediction results [122]. Copyright (2024) Elsevier. **f** Input/output format of RUL prediction based on LSTM many-to-one structure model [123]. **g, h** Compared with RUL prediction results of other models and MAPR evaluation indexes [123]. Copyright (2020) IEEE

While traditional ML methods offer baseline performance, DL approaches can capture more complex degradation dynamics. Ren et al. [117] combined autoencoders with DNN(ADNN) and proposed an integrated DL method for predicting the RUL of lithium-ion batteries. Its model structure of autoencoders is shown in Fig. 10a. Firstly, multi-dimensional feature extraction based on the autoencoder characterized battery health degradation, and then, the trained model predicted the remaining cycle life (Fig. 10b, c). The results showed that the RUL prediction curve was close to the actual data. The RMSE was 11.80%, and the accuracy rate was 88.20%. In contrast to the autoencoder-DNN hybrid methodology, graph convolutional networks (GCNs) are a type of neural network designed for processing graph-structured data and perform convolutions directly on graphs by aggregating information from a node’s neighbors and its own features [118]. This enables them to capture the local and global structure of the graph, as well as the relationships between nodes. The structure of the GCNs model is shown in Fig. 10d, and they have been widely applied to the prediction of the RUL [119–121]. Due to the limitations of traditional GCNs, such as not considering the connection between features and RUL, Wei et al. [122] constructed a conditional GCN using two classes of undirected graphs, considering feature-feature and feature-RUL correlations. The prediction results are shown in Fig. 10e. This method improves the RUL prediction performance, and the average RUL prediction RMSE is 3.484%.

Complementary to graph-based models, recurrent neural networks offer temporal modeling capabilities. Park et al. [123] proposed an LSTM-based RUL prediction technique with a multi-to-one structure (Fig. 10f), which adapted to different inputs, reduced parameters, and enhanced generalization. The results (Fig. 10g, h) showed that the MAPE of this model was controlled within the range of 0.47%-1.88%. Wang et al. [124] proposed a Bi-LSTM-AM model for online RUL prediction. The relative errors of the online RUL prediction for the six Li-ion batteries were 0.57%, 0.54%, 0.56%, 0%, 1.27%, and 1.41%, respectively. To provide a high-precision and high-efficiency basis for deep neural networks in RUL prediction, Ma et al. [125] developed a general physics-based model that can extract aging-related parameters from battery charging data, providing a high-precision and high-efficiency basis for DNN to predict the RUL. When only using data from one cycle, the mean absolute relative error (MARE) was 3.19%. Shifting focus to solid-state batteries, evolutionary algorithms offer unique optimization potential. Cao et al. [126] used symbolic regression (SR) on charge/discharge data of 12 solid-state lithium polymer batteries (cycle lives 71-213 cycles), achieving 87.9% test accuracy in predicting cycle life. Table 4 shows a comparison of the different RUL prediction method.

**Table 4:** Comparison of the different RUL prediction method

Method	Import	Prediction results	References
SVMR	V, I, C	MAE = 1.02% RMSE = 7.14%	[116]
ADNN	V, I, T	RMSE = 11.8%	[117]
GCN	V, I, T	RMSE = 3.484% MAE = 3.219% MSE = 15.80%	[122]
LSTM	V, I, T	MAPE = 0.47%-1.88%	[123]
Bi-LSTM-AM	/	relative errors = 0.54%	[124]
DNN	V, I, T	MARE = 3.19%	[125]
SR	/	test accuracy = 87.9%	[126]

### Battery Capacity Estimation

**Fig. 11:**
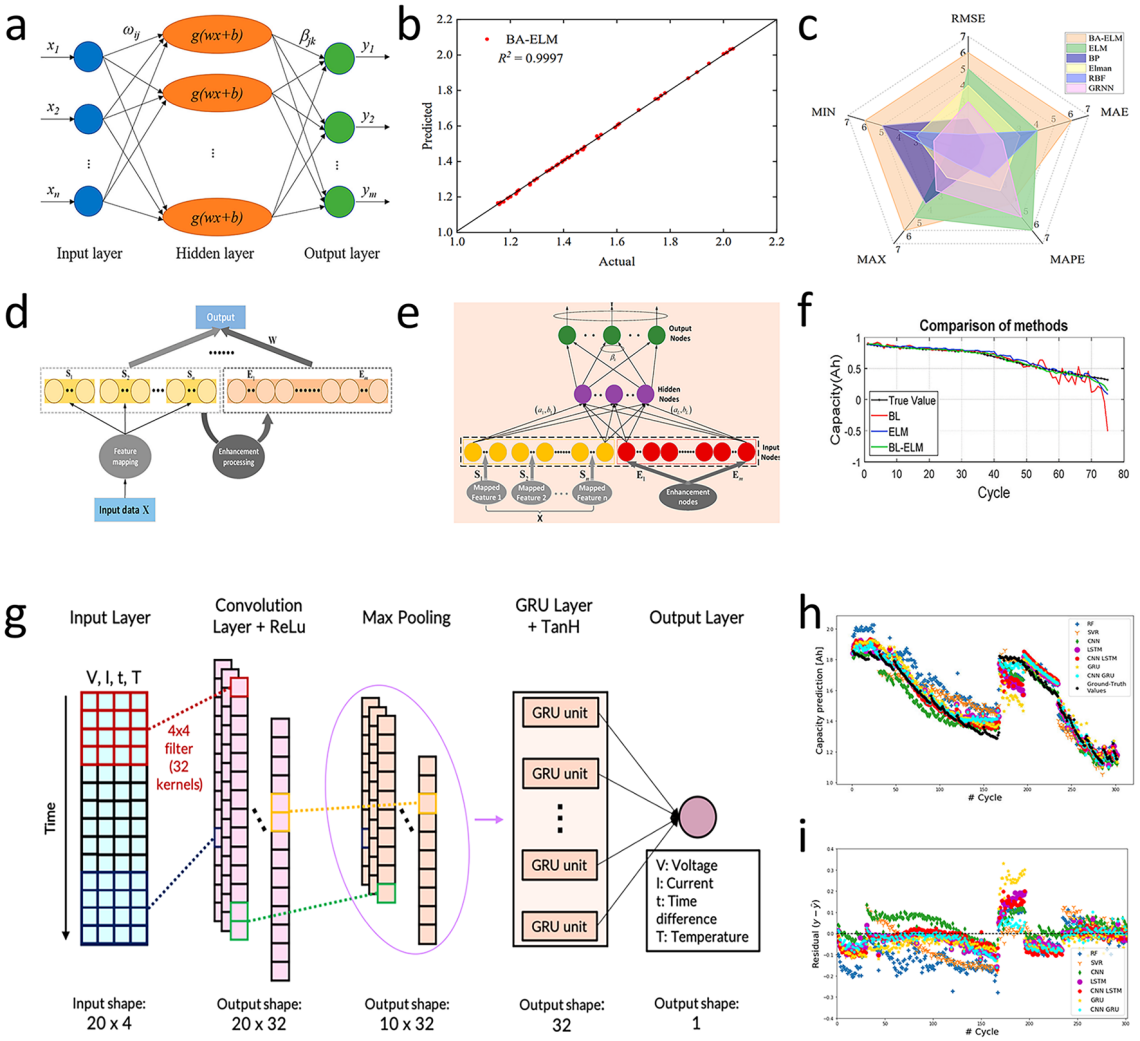
**a** ELM model structure [128]. **b** Actual capacity and predicted capacity are represented as scatter plots [128]. **c** Error radar map of each comparison model [128]. Copyright (2022) MDPI. **d** BL model structure [129]. **e** BL-ELM model structure [115]. **f** Capacity estimation results [129]. Copyright (2012) IEEE. **g** CNN-GRU model structure [133]. **h** Develop model capacity predictions [Ah], which are overlaid with ground-truth (black) extracted from the NASA dataset [133]. **i** Residual plot of capacity prediction [133]. Copyright (2020) IEEE

Battery capacity estimation can also break through the accuracy limitations of traditional methods [127]. Compared with traditional neural network methods, extreme learning machine (ELM) is a fast-learning algorithm for single-hidden-layer feedforward neural networks. It randomly initializes the input weights and biases of the hidden layer and analytically determines the output weights by solving a linear system. This approach significantly reduces the training time compared to traditional gradient-based learning methods, while still achieving good generalization performance (Fig. 11a). Ge et al. [128] used bat algorithm (BA) to optimize the connection weight and bias in ELM neural network and build the BA-ELM model. Then, the experimental data of BA-ELM, ELM, Elman, BP, radial basis function (RBF), and generalized regression neural network models are compared. The results show that the predicted value of BA-ELM model is consistent with the actual value (Fig. 11b), and the evaluation function can converge quickly. The RMSE of 0.5354% and MAE of 0.4326% are the smallest of the six models (Fig. 11c). Ma et al. [129] introduced the idea of broad learning (BL) (Fig. 11d) and constructed the broad learning-extreme learning machine (BL-ELM) model, whose structure is shown in Fig. 11e. The results indicated that the BL-ELM method can not only ensure the accuracy of estimation and prediction (Fig. 11f) but also save a significant amount of time. Shen et al. [130] proposed a DL-based capacity estimation method that combines the concepts of transfer learning and ensemble learning and get a DCNN with ensemble learning and transfer learning (DCNN-ETL). It can achieve good results even when there is less training data.

Beyond the traditional neural network optimization and combined learning methods, Vakharia et al. [131] proposed an interpretable artificial intelligence (Ex-AI), through training based on 6 input features. After exploring various single and combined models, they developed three DL models (Stacked LSTMs, GRU Networks, and Stacked Recurrent Neural Networks) to predict battery discharge capacity. The results show that the superposed LSTMs model has the best prediction effect, with RMSE of 0.04, MAE of 0.60, and MAPE of 0.03. Oyucu et al. [132] used AdaBoost, Gradient Boosting, XGBoost, LightGBM, CatBoost, and ensemble learning models to predict lithium-ion battery discharge capacity, aiming to improve energy-storage-system cost-effectiveness in large-scale applications. The results indicated that the LightGBM model had the lowest prediction MAE (0.103) and mean square error (MSE) (0.019), demonstrating the strongest correlation. After exploring various single and combined models, Crocioni et al. [133] compared different ML algorithms for estimating lithium-ion battery maximum releasable capacity. After tenfold training and testing, neural networks outperformed random forest (RF) and SVM (Fig. 11h, i). In particular, the CNN-GRU (Fig. 11g) was better than other neural network architectures, with the maximum values of RMSE and MAE being 0.0488 and 0.0414, respectively. RVM is a Bayesian-based sparse supervised learning algorithm that can address SVM deficiencies [134, 135]. Guo et al. [136] utilized the PCA method to reduce the feature dimension during the model training process and combined PSO and RVM to find the optimal hyperparameters. The proposed PSO-based RVM framework can limit all relative errors within 2% under the working temperature range of 24-43 °C. Table 5 shows a comparison of the different capacity estimation method.

**Table 5:** Comparison *f* the different battery capacity estimation method

Method	Import	Prediction results	References
BA-ELM	T_I_, T_V_, ∆V _charge_, T _total, discharge_, ∆V _discharge_, ∆Temp _discharge_	RMSE = 0.5354% MAE = 0.4326	[128]
BL-ELM	V, I	RMSE = 2.88%	[129]
DCNN-ETL	V, I	RMSE = 1.503% Max Error = 9.505%	[130]
Stacked LSTMs		RMSE = 0.04 MAE = 0.60 MAPE = 0.03	[131]
LightGBM	V, I, T Cycle Index Discharge Capacity	MAE = 0.103 MSE = 0.019	[132]
CNN-GRU		RMSE = 0.0488 MAE = 0.0411	[133]
RVM	V, I, T	Error < 2%	[136]

### Evaluation Index

In the classification task of interfacial stability for solid-state batteries, accuracy quantifies the proportion of correctly predicted samples relative to the total, reflecting the model’s overall discriminative capability. This metric is reliable under balanced class distributions (e.g., stable/unstable interfaces each at 50%), but requires complementary evaluation with precision and recall for imbalanced datasets (e.g., failure samples ≤ 5%).

In the research of material property prediction, the evaluation of regression models should be closely centered around the physical significance of the target variables and engineering requirements. The following indicators quantify the prediction capabilities of the models from multiple dimensions.

RMSE measures the average magnitude of the errors in a set of predictions. It is a widely used metric in regression analysis to assess the accuracy of prediction models. RMSE gives a sense of how far, on average, the predictions are from the true values, with the advantage that it penalizes larger errors more severely due to the squaring operation. For instance, if RMSE = 10, it can be considered that the regression results deviate from the true values by an average of 10 in magnitude. In addition, MAPE is used to evaluate the accuracy of a forecasting method in percentage terms. It shows the average percentage difference between the predicted and actual values. A MAPE of 0% indicates a perfect prediction model, while values above 100% suggest a poor-performing model. However, it is important to note that when the true value *y*_*i*_ = 0, the MAPE formula cannot be applied due to division by zero. Moreover, MAE calculates the average of the absolute differences between the predicted and actual values. MAE is straightforward to interpret as it has the same unit as the original data, directly representing the average gap between predictions and true values. Compared to MSE or RMSE, MAE is more robust to outliers because it does not square the errors. Furthermore, MSE computes the average of the squares of the errors. MSE amplifies larger errors due to the squaring operation. RMSE is simply the square root of MSE, which makes RMSE more interpretable in terms of the scale of the original data.

## Conclusion and Perspective

**Fig. 12:**
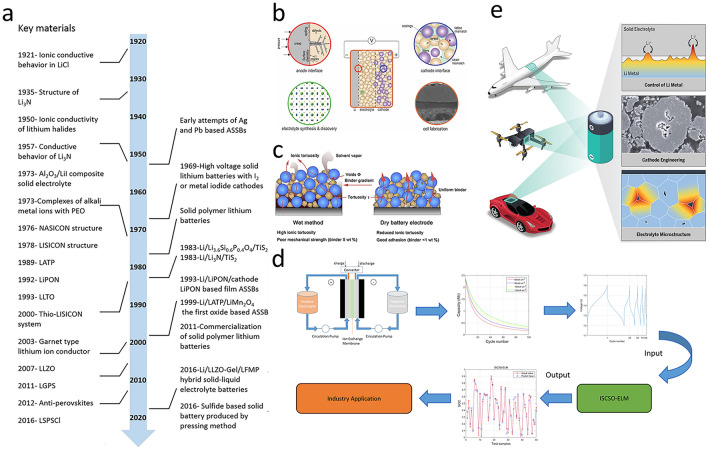
**a** Timeline of ASSBs key material and technology development [137]. Copyright 2019 American Chemical Society. The content in the figure is based on the reference [138–159]. **b** Schematic of the potential scientific challenges impeding the development of solid-state batteries [160]. Copyright 2020 The Author. **c** Solid-state battery electrode manufacturing challenges [161]. Copyright 2022 Elsevier. **d** SOC estimation process by ISSO-ELM method [162]. Copyright 2023 American Chemical Society. **e** Application prospect of solid-state battery [163]. Copyright 2023 American Association for the Advancement of Science

Reviewing the development history of key materials and technologies of ASSBs (Fig. 12a) [137], traditional research and development methods have led to extremely slow progress. Each new material requires extensive experimental tests and theoretical analyses. From the optimization of the material synthesis process to the research on its compatibility with electrode materials, each step consumes a great deal of time and resources. Currently, ASSBs face numerous intractable issues across multiple key aspects. Figure 12b [160] reveals four major challenges: the anode interface, the cathode interface, the synthesis and discovery of electrolytes, and battery manufacturing. Additionally, solid-state battery (SSB) electrode manufacturing has significant problems. In SSB electrodes, ion transport mainly relies on solid–solid contact (Fig. 12c) [161], which severely restricts ion transport efficiency. As electrode thickness increases, ion transport resistance rises, leading to a sharp decline in material performance.

With the rapid development of artificial intelligence technology, it not only provides new ideas for addressing the above-mentioned challenges and accelerates the material screening process, but also enables more accurate evaluation of battery performance parameters. For example, Xiao et al. [162] applied AI to vanadium redox flow batteries, establishing an electrochemical model to determine ion concentrations. They combined the extreme learning machine (ELM) with the improved sand cat swarm optimization algorithm (ISCSO-ELM) for state-of-charge (SOC) estimation. Experiments showed this approach outperforms traditional methods in predicting battery SOC, facilitating precise battery management (Fig. 12d). If breakthroughs are achieved in engineering battery components, new electrode materials and SSEs are developed, the interface compatibility issues are resolved, and no additional pressure is required to maintain the integrity of the interface during manufacturing, the cost and difficulty of use can be reduced. With their excellent performance, ASSBs will play a huge role in multiple fields (Fig. 12e) [163]. Particularly in the new energy vehicle field, ASSBs could significantly increase driving range and charging speed and reduce weight and cost. In aviation, their high energy density and safety can improve the flight range and flight performance, reduce maintenance costs, and contribute to the innovation of the aviation industry. In the fields of smart grids, portable electronics, and wearable devices, ASSBs can serve as energy storage devices and long-lasting power sources. Therefore, it is of great importance to fully utilize AI to empower solid-state batteries and accelerate this process.

In this review, we explore how ML and DL have made significant progress in solid-state batteries, highlighting that these technologies enable more accurate prediction and efficient management, optimize battery performance and extend battery life, and are already widely used in traditional lithium batteries. While these algorithms have shown great potential in the material exploration of solid-state batteries, mining potential associations in massive material data to identify potential electrolyte materials and expand the selection of materials, there is still much room for further integration between them in the comprehensive evaluation of their performance. At the same time, there are still many challenges that prevent their wider application in solid-state batteries:

1. *Exhibit Poor Portability*: Different electrolyte systems or performance prediction tasks have different requirements for the architecture and parameters of ML models. Current models exhibit limited reusability and suboptimal portability. When new problems arise, it is necessary to rebuild the model and process the data again, resulting in a waste of resources. Future research should focus on designing universal algorithms and standardized model frameworks and building a data sharing platform. Through cross-disciplinary cooperation, develop adaptable model structures to achieve rapid adjustment and reuse of models in different solid-state battery systems and improve research and development efficiency.
2. *High-Dimensions Small Sample Data*: The internal environment of the battery is complex [164], resulting in a large number of dimensions in the battery’s internal data. For ML algorithms, whether regression or classification, a large number of dimensions and a small number of samples will cause the model to be easily affected by noise interference and the generalization ability to deteriorate. To address this problem, dimensionality reduction techniques such as PCA [165, 166], linear discriminant analysis (LDA) [167, 168], and feature selection [169, 170] can be used to extract key features. At the same time, the dataset can be expanded through generative adversarial networks (GAN) [171–173].
3. *Issues Regarding Data Quality and Quantity*: When using ML and DL for prediction, it is necessary to ensure the quality and quantity of data. Currently, there are deficiencies in reflecting the real structure of materials, mining feature relationships, and retaining physical and chemical details. Advanced material characterization techniques can be combined to obtain accurate data, a neural network based on physical knowledge can be constructed, and transfer learning can be applied to adapt to different material systems, so that the generalization ability and prediction accuracy of the model can achieve good results on different materials.
4. *The Comparison of Different Models Encounters Formidable Obstacles*: In the article of different authors, due to differences in data, parameters, algorithms, and hardware conditions, it is difficult to cross-compare different models. The complexity of composite algorithm models is high, and the controllability is weak. Their actual effects and robustness need further verification. In future research, a unified evaluation standard and dataset can be established, data records can be standardized, and the reproducibility of papers can be improved. At the same time, modular design and stability analysis of composite algorithm models can be carried out, the interaction mechanism between modules can be optimized, and the overall controllability and robustness of the model can be improved. Methods such as cross-validation and ensemble learning can be adopted to integrate the advantages of multiple models and improve the adaptability and stability of the model in different datasets and application scenarios.
5. *The code is not publicly available*: Since the inputs and parameters of the ML models developed by different researchers are different, the differences in the data processing stage also affect the performance of the model. The non-public code makes it difficult to reproduce and fairly compare the research, hindering the improvement of the methods proposed by different researchers. We advocate that researchers disclose the source code and related data of their models, establish an open-source battery research code library and platform, standardize code writing and data management, optimize the code through community collaboration, improve the transparency and reproducibility of the research, and promote the technological progress of the entire field.
6. *The non-interpretability of the black-box model*: ML is often regarded as a “black box” operation because the mapping relationship from input data to output results is complex and difficult to explain intuitively [174, 175]. The internal mechanism is complex and lacks a clear association with physical and chemical principles. To address this problem, multiple measures can be taken: Firstly, apply explainable artificial intelligence (XAI) technique [176, 177], such as determining the importance of input features to the output through feature importance analysis and using model visualization techniques to display the decision-making process of the model; secondly, combine domain knowledge, integrate physical and chemical principles into the model construction as constraints, and promote the knowledge interaction between domain experts and ML researchers; thirdly, add validation data during the model learning process to evaluate performance, and after the output, judge the rationality of the result through rules and additional calculations, adjust or re-evaluate the model for unreasonable outputs, thereby improving the reliability and interpretability of ML in battery research and other fields.

Building on the advancements in AI-driven material screening and performance evaluation discussed in this review, the next frontier for solid-state battery innovation lies in harnessing emerging AI paradigms to address unresolved challenges and unlock new design possibilities. Future research should prioritize the following directions to accelerate commercialization:

1. *Generative Adversarial Networks for Novel Material Design*: Generative AI models such as GAN or variational autoencoders can generate entirely new material structures, providing innovative ideas for the design of solid-state battery materials. These models can generate unique material structures with distinct properties based on learning the distribution of existing material data, thus expanding the possibilities for material research and development.
2. *Reinforcement Learning for Multi-Parameter Battery Optimization*: Reinforcement learning algorithms can be used to optimize multiple parameters of solid-state batteries, such as electrode composition, electrolyte thickness, and battery operating temperature. Reinforcement learning can explore within a complex parameter space to find the optimal combination of parameters, thereby enhancing the energy density, power density, and cycle life of the batteries.
3. *Convolutional Neural Networks for Electrode-Electrolyte Interface Analysis*: AI image recognition and analysis techniques (such as convolutional neural networks) can be used to conduct microscopic structural analysis of the electrode/electrolyte interfaces in solid-state batteries, identifying interface defects, phase boundaries, and chemical reaction products. Through learning from a large amount of interface image data, AI can quickly and accurately evaluate the interface state, providing guidance for interface engineering.
4. *AI-Enabled Adaptive Battery Management Systems*: An AI-based battery management system can adaptively adjust the charging and discharging strategies according to the real-time state of the battery and the working environment, optimizing the battery’s usage efficiency and lifespan. Through reinforcement learning and real-time data feedback, the system can continuously learn and adjust the control strategies to adapt to different working conditions.
5. *Multi-scale modeling with AI integration and explainable AI*: As for emerging research frontiers, one area that holds great promise is the integration of multi-scale modeling with AI. Combining atomistic, mesoscopic, and macroscopic models with ML algorithms can provide a more comprehensive understanding of solid-state electrolyte behavior across different length and time scales. Another frontier is the development of XAI for solid-state electrolyte research. XAI algorithms can help researchers understand the underlying reasons behind model predictions, facilitating the translation of AI-generated insights into practical material design strategies.
6. *Integration of novel solid-state electrolytes with AI*: The integration of novel solid-state electrolytes with AI methodologies indeed has the potential to drive further advancements. For example, AI could be used to fine-tune the composition and structure of the h-PAN@MOF network, predicting how changes in the MOF crystal structure or the PAN fiber network would affect ion conductivity, mechanical strength, and interfacial stability [178].

## Data Availability

No primary research results, software, or codes have been included, and no new data were generated or analyzed as part of this review.
